# Supplementary figures and images for: The impact of clade B lineage 5 MERS coronaviruses spike mutations from 2015 to 2023 on virus entry and replication competence
Source: PLoS Pathog. 2026 Feb 3;22(2):e1013336. doi: 10.1371/journal.ppat.1013336 (PMC12904574; doi:10.1371/journal.ppat.1013336)

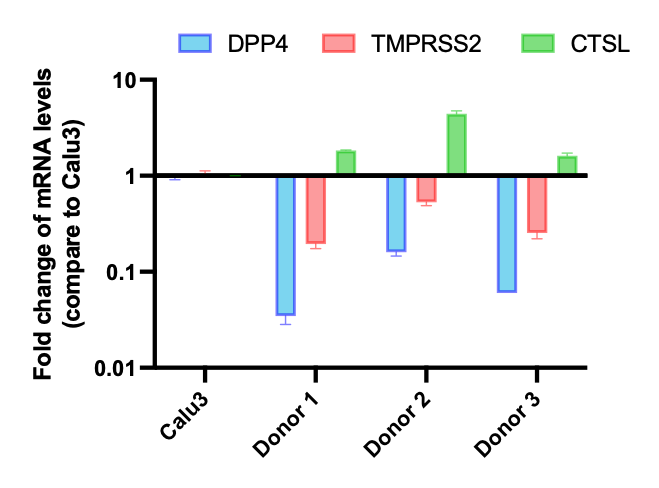

Supplement: S1 Fig — Three independent donors of alveolar epithelial cells were extracted for cellular RNA. RNAs were reverse transcribed and quantified for expression of DPP4, TMPRSS2 and CTSL. Expression levels were compared by ΔΔCt method across samples with Calu3 cells as reference. Error bars indicate mean with SD. (TIFF) [file ppat.1013336.s001.tiff]

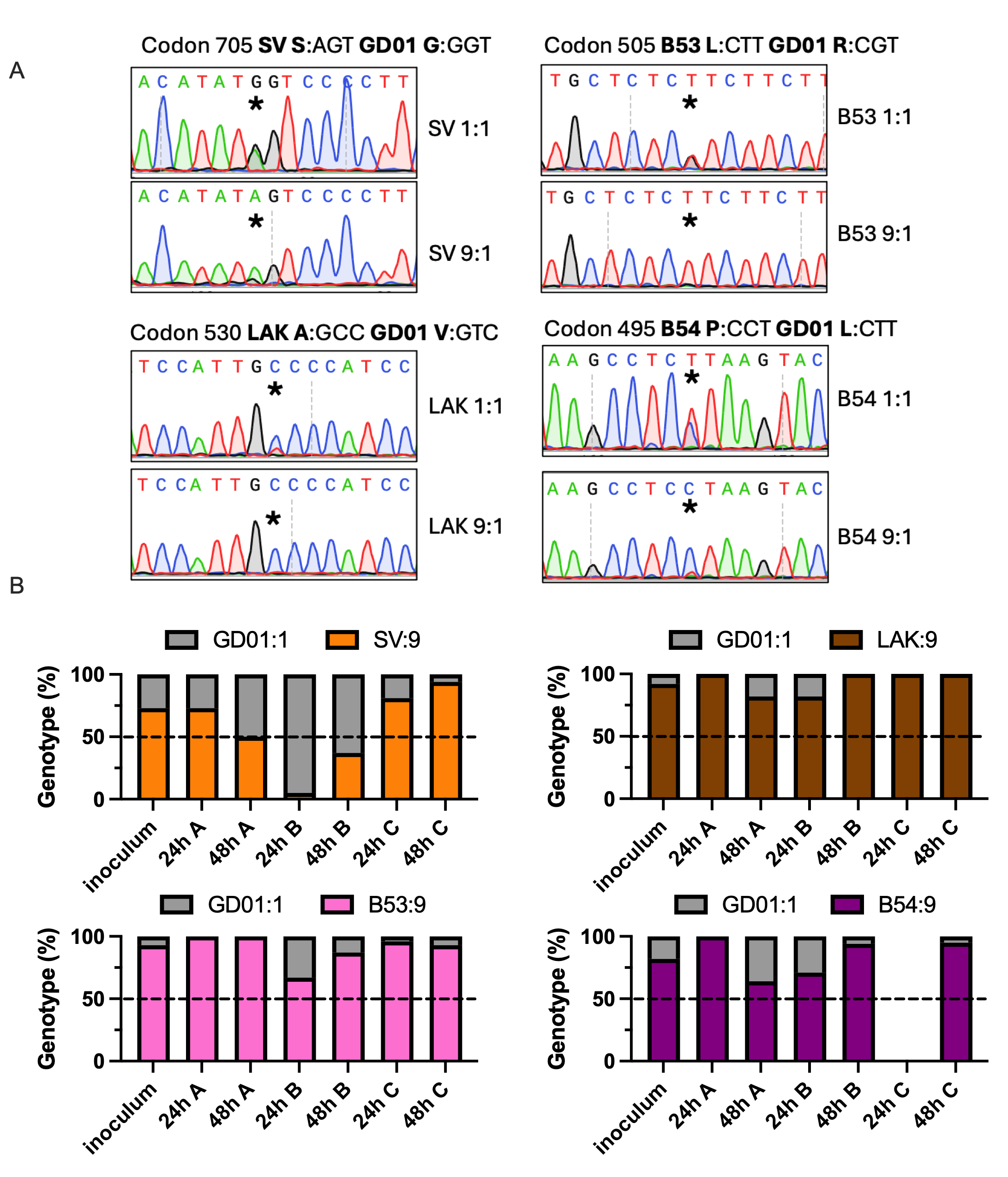

Supplement: S2 Fig — (A) Peak information from sanger sequencing of inoculum genotypes. The specific mutation for genotyping of each spike mutant is indicated, and the corresponding nucleotide is marked as asterisk (*). (B) Genotyping result for inoculum at ratio of 9:1 of spike mutant vs 2015/GD01. The 24 timepoint of donor C in B54 did not yield successful PCR amplification. (TIFF) [file ppat.1013336.s002.tiff]

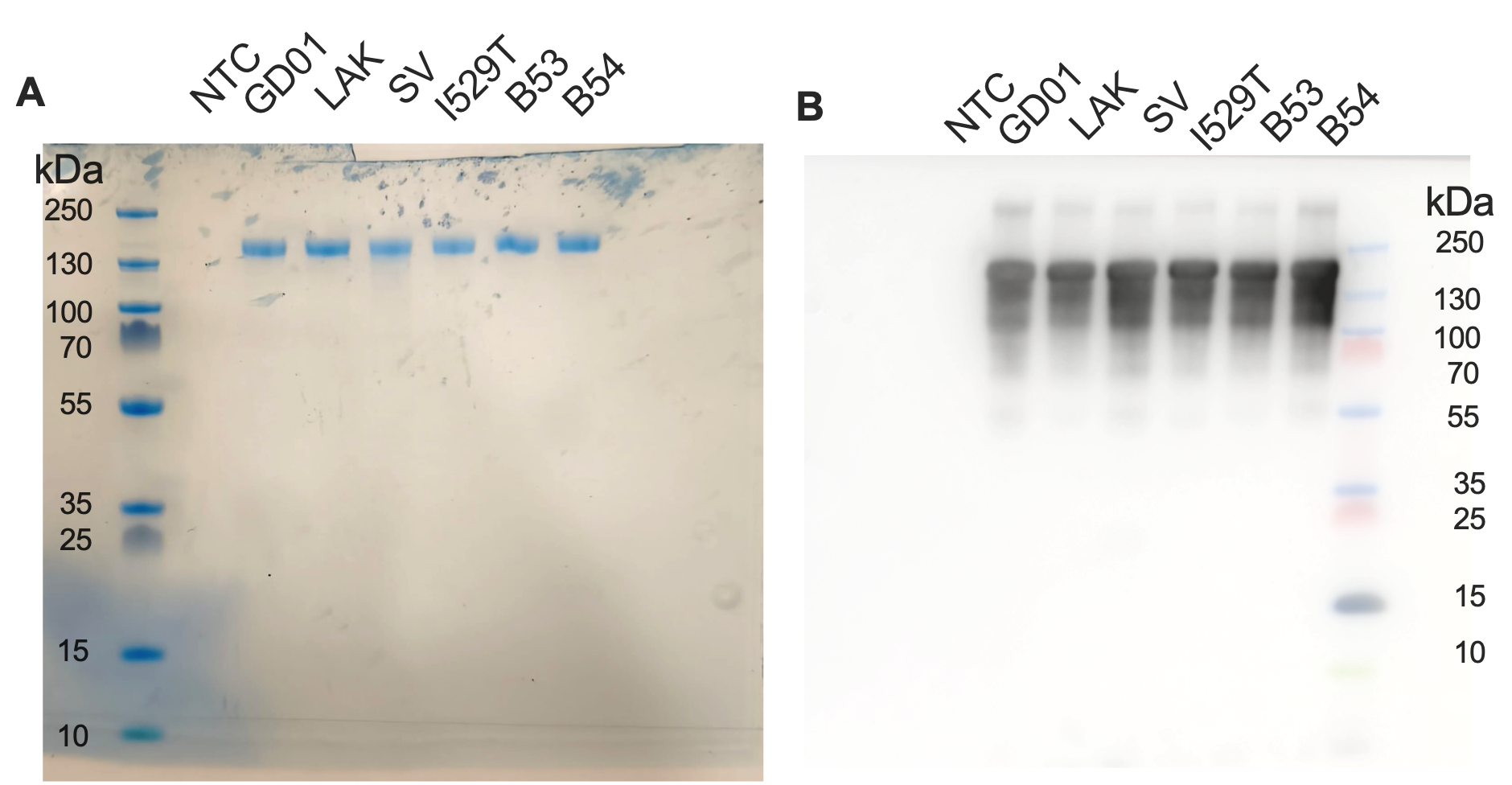

Supplement: S3 Fig — (A) 1ug of purified S1-Fc proteins were loaded on a denaturing 4–12% Bis-Tris SDS-PAGE. The gel was stained with Coomassie Brilliant Blue R-250 staining solution. Position and sizes of the ladder are indicated. Non-transfected control (NTC) was the negative control to validate no unspecific Fc proteins were purified from the Protein A/G column. (B) Western blot of S1-Fc proteins on a denaturing 4–12% Bis-Tris SDS-PAGE. S1-Fc proteins were stained by primary MERS-CoV Spike Antibody (40069-T62, Sino Biological) and secondary anti-rabbit IgG. The S1-Fc band shared the same position as in the Coomassie Blue staining. (TIFF) [file ppat.1013336.s003.tiff]

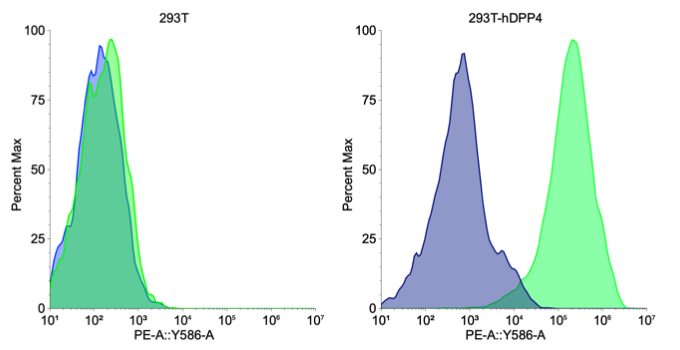

Supplement: S4 Fig — 293T and 293T-hDPP4 cells were stained by PE-conjugate anti-DPP4 antibody (10688-MM05-P, Sino Biological) (Color in Green). Mouse IgG1 kappa isotype PE served as background control (14-4714-82, Thermo) (Color in Blue). (TIFF) [file ppat.1013336.s004.tiff]
