# Supplementary material for: The impact of clade B lineage 5 MERS coronaviruses spike mutations from 2015 to 2023 on virus entry and replication competence
Source: PLoS Pathog. 2026 Feb 3;22(2):e1013336. doi: 10.1371/journal.ppat.1013336 (PMC12904574; doi:10.1371/journal.ppat.1013336)
Supplement: Attachment — Submitted filename: Response to Reviewers MP.docx [file ppat.1013336.s007.docx]

**Responses to Reviewers' Comments:**

We thank the Editor and the Reviewers for constructive comments on our manuscript. We have carried out additional experiments to address some of the reviewer comments. We now provide the revised manuscript and a point-by-point response to each of the reviewer comments, below.

Reviewer's Responses to Questions

**Part I - Summary**
Please use this section to discuss strengths/weaknesses of study, novelty/significance, general execution and scholarship.

Reviewer #1: The manuscript bySo and colleagues describe phenotypic analyses of MERS-CoV clade B lineage 5 viruses with a particular focus on the spike gene and it's impact on virus fitness. The authors start with the hypothesis that the decline of reported MERS cases since 2018 may be due to phenotypic changes. To address this question, the authors have first generated a set of lentivirus MERS-spike pseudotype viruses contain spike genes representative for MERS-CoV evolution based on the reported MERS-CoV sequences in public databases. As basis for comparison the ancestral clade B lineage 5 2015/GD01 stain was used. Representative sequences were derived from clade B lineages detected in the time period of 2013 to 2023. The lentiviral pseudotypes were used to assess spike-mediated cell entry in Vero and Calu3 cells (for endosomal vs pasmamembrane entry routes). While early clade B lineage 2, 3, and 4 spikes did not show any differences in virus entry compared to 2015/GD01, spikes derived from 2019 sequences showed reduced entry in both, Vero and Calu cells. Notably, spike genes representing more recent variants from 2023 did again not show any difference in entry compared to 2015/GD01. To analyze possible spike-mediated phenotypic changes in MERS-CoV infections, the authors employed reverse genetics to clone the different spike genes into the backbone of 2015/GD01. Again infections were don in Vero and Calu3 cells and overall viral plaque phenotypes and growth kinetics corroborated the lentiviral pseudotype date with viruses contain the 2019 spikes showing slightly reduced growth, while viruses containing the 2023 spikes show similar growth compared to 2015/GD01.
Overall, the described phenotypic analysis of MERS-CoV strains that represent MERS-CoV evolution in camels is of great importance in the context of pandemic preparedness and to assess if phenotypic changes can be observe that may increase or reduce the risk of human infection. However, the study do not use the full spectrum of experimental system that are available. The rather minor phenotypic differences and the exclusive use of cell lines limit the impact.

Response: We thank the reviewer for the interest of our study and providing constructive experimental comments.

Specific comments:

Note: All line numbers mentioned below refer to the clean manuscript file.

1. A logical extension of this study would be the assessment of viral phenotypes in primary culutres, such as nasal, bronchial and alveolar epithelial cultures and organoids. The authors have most of these experimental systems available. It would be of interest if replication kinetics, target cell tropism and possibly host cell responses may differ.

Response: We extended the phenotype experiments in primary nasal and alveolar epithelial cells as suggested (Fig.4). We selected the 2019 and 2023 sequences for a further comparison with GD01. We found that both 2019 spikes (LAK and SV) showed concordant phenotypes as shown in Calu3 cells while 2023/B53 remained showing similar replication kinetics as GD01. The 2023/B54 showed a distinct reduced kinetics phenotype in both nasal and alveolar cells, which was not seen in Calu3 and VeroE6-TMPRSS2 cells previously. We discussed that this difference could be attributed to the additive effect of differences in expression of DPP4, TMPRSS2 and CTSL between these cell types that we demonstrated (lines 304-7). We demonstrated mutations at the NTD and S2 domain of 2023/B54 may also be contribution factors.

Since the revised manuscript now report an additional reduced replication phenotype in 2023 spike sequences, we amended the conclusion of the study accordingly and now report spike mutations from 2019 and 2023 can demonstrate reduced replication phenotype in human cells (Line 26-29; 42-43; 284-86).

2. To specifically compare viral fitness, competition assay may be useful. In particular if primary airway cultures can be employed.

Response: We performed competition growth assay in primary alveolar epithelial cells as suggested (Fig.4). We prepared inoculum of ratios of 1:1 and 9:1 mutant vs GD01 and infected to individual wells from three separate donors. For 1:1 mutant vs GD01, we observed mutant viruses LAK, SV and B53 were outcompeted by GD01 in 2/3 and 3/3 (B54) donors. The result supported the replication phenotypic trend as shown in primary alveolar cells (Fig.4B). The variable outcome in the competition assay (2/3) with LAK, SV and B53 could be due to donor variation. For 9:1 mutant vs GD01, we did not observe dominance of GD01, except with one donor and with 2019/SV. We suggest that the short duration (48h) of the competition assay at a very challenging 9:1 ratio probably does not allow GD01 virus to manifest genotype dominance.

3. The authors may want to include lack of reporting of MERS cases during the COVID pandemic as one additional potential reason for the decline in reported MERS cases.

Response: We agree with the reviewer that the true incidence as well as reporting of MERS cases may have been affected by the response to the COVID-19 pandemic. But this does not explain the continued low levels of cases for 2023-25. An upcoming WHO “Middle East respiratory syndrome: global summary and assessment of risk” report due our in January 2026, which the one of the authors (MP) has contributed to states that reduced surveillance is unlikely for the observed reduction of cases in the Arabian Peninsula, “as testing volumes remain high in countries with the highest incidence, including through sentinel surveillance systems and the use of sensitive case definitions for identifying suspected cases”. We have referenced this report in press.

Therefore, in lines 374-79 we state that “While public health and social measures would be expected to reduce incidence of most respiratory virus diseases, including MERS, these are no longer in place post 2022 and cannot explain the continued low level of reported MERS cases. Levels of testing for MERS-CoV in many countries in the Arabian Peninsula remains high and lack of testing is unlikely to explain that the recent reduced MERS cases.”

Reviewer #2: MERS-CoV is an important emerging coronavirus with zoonotic potential and a high mortality rate in humans. This study seeks to understand the recent decrease in human cases of MERS-CoV by examining spike mutations which have arisen in recent years. They also utilize a Golden Gate cloning strategy for efficient synthesis of MERS-CoV infectious clones, which could be of use in the field. This study provides interesting preliminary data utilizing the sequences of MERS-CoV genomes available and generates many interesting questions to address in the future. There are a few areas where the paper could be strengthened and the authors should be careful with their language on how their findings connect to the recent decrease in human cases. Overall, the study is very well written and clear. The figures are organized and well put together.

Response: We thank the reviewer for the interest of our study and providing constructive comments to refine our manuscript.

Reviewer #3: In the paper, So et al discuss the impact of substitutions that have appeared in strains of MERS on viral entry and replication. They employ both pseudotyping and live virus approaches to assess the contribution of mutations. While the data is interesting, the authors do not provide a comprehensive picture of the effect of the studied substitutions nor mechanism, leaving many open questions.

Response: We thank the reviewer for the interest of our study and providing constructive experimental comments.

**Part II – Major Issues: Key Experiments Required for Acceptance**
Please use this section to detail the key new experiments or modifications of existing experiments that should be absolutely required to validate study conclusions.

Reviewer #1: The inclusion of primary airway cultures is important

competition experiments to corroborate growth kinetics results.

Response: We performed the reviewer’s experimental requests and provided responses as mentioned above.

Reviewer #2: No responses needed.

Reviewer #3: For the all the S studied in using the lentiviral pseudotyping approach, authors should purify and analyze the incorporation/presentation of S on the pseudovirions by immunoblot. They need Spike westerns and functional DPP4 usage experiments for all these mutants to understand processing and S1/S2 status on pseudoparticles.
Concerning the mechanism of entry, the authors investigated if the substitutions detected have an impact on the pathway of entry. This was assessed using Camostat and E64d, inhibitors of TMPRSS2 and Cathepsin L, respectively. In addition to this, the authors could consider comparing cell lines with high and low surface expression of TMPRSS2, such as HuH7 and Caco2/Calu3.

Response: We performed western blotting to confirm the spike cleavage and incorporation as suggested by the reviewer and results are shown in Fig2E. Overall, the spike cleavage among the spike mutants were consistent with GD01, as shown by the similar ratios of band intensities of full spike and S2. The spike incorporation into virions were determined by the total band intensities of full spike and S2 per the band intensity of p24, a housekeeping lentiviral protein expressed in the lentivector. We found a consistent spike incorporation among the spike mutants relative to GD01 without significant differences. Now in text amended lines 160-63.

We have repeated the entry pathway experiment in Huh7 and Caco2 cells using pseudoviruses as suggested. We performed RNA expression analysis and flow cytometry to validate the expression DPP4, TMPRSS2 and CTSL among different cell lines (293T, Calu3, Caco2, Huh7, Vero and VeroE6/TMPRSS2) (Fig5C). Based on the expression profile, we confirmed Huh7 cells were low in TMPRSS2 surface expression, whereas Caco2 cells were relatively high. We next performed entry inhibition experiments in these cells by pretreated cells with Camostat (to block TMPRSS2 entry pathway), E64d (to block endosomal CTSL entry pathway) and both drugs together (to block both pathway) (Fig5D). We found no substantial changes in entry pathway preferences among these spike proteins in both cells, indicating the entry pathways remained unaltered due to these mutations.

Figure 2. There is no data for some mutations, e.g. V26L or Q914K. Please add a full breakdown of individual point mutants.

Response: We performed individual point mutants for 2019/LAK as suggested and confirmed the V530A was the determinant mutation for the reduced cell entry (Fig2c).

Figure 3: The main results from Figure 2 are not taken forward to Figure 3. Why have the authors not made the individual point mutations (e.g. V530A) in live virus?

Response: In Figure 2, we identified the 2019 spike proteins were reduced in cell entry, whereas recent 2023 spike proteins were not showing major changes in cell entry. We therefore put forward the findings by generating replication-competent recombinant viruses to study the phenotypes in the MERS-CoV backbone. We aimed to generate full-spike chimeric recombinant viruses to validate our findings using pseudoviruses in Figure 2 (A, B and D). Overall, we created six novel recombinant viruses, that covered spike proteins with normal cell entry (2018/T424I), reduced entry control (2015/I529T), reduced entry (2019/LAK, 2019/SV) and two most recent representative spike sequences (2023/B53 and 2023/B54).

We did not generate individual point mutation viruses because the priority of this study was to compare the spike phenotype across the sampling years, rather than identifying specific mutations that were responsible for reduced cell entry and replication. We agree that finding the specific phenotype determinant spike mutations will also be of scientific importance to understand the MERS-CoV biology. Given the extensive work to generate extra combinations of recombinant viruses and the coverage limitation of this manuscript, we would like to investigate these specific spike mutations in future studies.

**Part III – Minor Issues: Editorial and Data Presentation Modifications**
Please use this section for editorial suggestions as well as relatively minor modifications of existing data that would enhance clarity.

Reviewer #1: see point 3 in part I

Response: We provided the responses in part I as mentioned above.

Reviewer #2: 1. Abstract line 38-40 and discussion line 325-327: While the data my suggest that the spike mutations are not driving the decrease in human cases, viral entry and replication were only examined in 2 tissue culture cell lines. This is not sufficient to conclude that mutations in the spike are or are not contributing to the number of human cases. The authors should soften their language in these places to reflect this.

Response: We agree and removed the relevant sentences. With the new results from 2023 spike sequences showing reduce replication and lower viral fitness in primary respiratory epithelial cells, the main conclusion of this study is the identification of the reduced cell entry and replication impact on MERS-CoV due to spike mutations in recent years (2019-2023).

In the revised manuscript, we avoided the direct linking of spike mutation to the reduced zoonotic cases. Instead, we aimed to deliver our findings may provide novel insights to understand the recent MERS epidemiology (line 33-34; 284-89; 364-66).

2. The amount of spike incorporation into lentivirus pseudotypes should be reported, perhaps via ELISA quantificaion. It is possible that the decrease in entry from the 2019 strains could be due to decreased spike incorporation especially given one of the mutations seen is in the TM.

Response: We validated the spike incorporation into lentivirus pseudotypes as suggested by another reviewer using western blotting (Fig.2E). We confirmed spike incorporation of mutants remained no significant changes as compared to GD01 spike.

3. Plaque sizes of infectious clones should be quantified and reported in paper.

Response: We have added the quantified plaque size data in Fig3D as suggested. We reported the plaque size findings in the result section (line 176-79).

4. Figure 3 – please indicate unit of time for X axis in Figure 3 E, F, G, and H in the figure legend.

Response: We have added the unit of time in Fig3 E-H as suggested.

Reviewer #3: In the discussion, the authors talk about SARS-CoV-2 S2-specific antibodies, and their potential impact for cross-protection against MERS. There is a recent study that shows no cross-immune response against MERS of sera from SARS-CoV-2 infected individuals [1], while paper suggests that anti-S2 antibodies cross react against MERS and its related viruses [2]. They should be mentioned in the discussion
It has been reported that MERS lineage 5 is less susceptible to IFN response. I wonder if replication studies should therefore be performed in Vero cells, which are defective for type-I IFN. I would suggest validating the results in human cell lines permissive to MERS, in addition to Calu3. Importantly, it has been showed that lineage 5 MERS increase in viral replication was dependent on reduced induction of immune genes [3], suggesting that evolution of MERS impacts replication at the level of the innate immune response antagonism, and less at the entry step of the viral cycle. The authors briefly mention this, but I think it should be better and more extensively addressed. Overall, the study is interesting and contributes to our understanding of MERS evolution and its role during entry, but I believe the paper addresses only part of the story, with limited results, and should also present data concerning more detailed unravelling of the replication step of the virus, considering that the authors develop and talk extensively about the Golden Gate approach they adapted to the virus.
For figure 2, it was not clear to me what the different colours of the bars represent. After looking at figure 3, I assumed that they were the different strains. I think the colour code should be mentioned in the legend of the figure.

Response: We thank the reviewer for providing additional references relevant to this topic. We have mentioned that SARS-CoV-2 infection or vaccination can elicit spike S2 domain binding antibodies. The citation we used studied post SARS-CoV-2 infection with different combinations of vaccination platforms and priming scenarios. The study reported the presence of cross-reactive antibodies towards S2, but not S1 domain of MERS-CoV. The S2 binding antibodies did not showed neutralization using ppNT assay. We believe the citation covered similar findings as mentioned in the two references.

We have added more discussions related to the enhanced IFN antagonism in lineage 5 MERS-CoV, the current circulating lineage. We mentioned that other genetic changes outside spike gene could also contribute to the reduced human cases. Currently, there is no comprehensive phenotype data of 2023 MERS-CoV isolates. We believe possibilities remain for spike mutations, other genomic mutations, or both mutations together, to contribute to the observed MERS epidemiology (line 354-59).

We have now provided a clear color legend in Fig 2A to indicate the years of spike sequences and mentioned in the figure description.

Minor comments:
What is the rationale for looking at entry in Veros? Please examine by flow to understand DPP4 expression relative to Calu3s? Can the authors find more human cell lines expressing DPP4 to do this work?

Response: We performed entry experiments in Vero cells to test for cell entry via the endosomal pathway. We have performed surface expression analysis for DPP4 and TMPRSS2 in more human cell lines as suggested (Fig5C). The expression data confirmed Vero cells as a model of endosomal entry as it expresses less TMPRSS2 than Calu3. We found Calu3 and Vero cells express similar levels of DPP4.

Line 133: NTD is repeated twice

Response: We have corrected the typo.

Line 179-180: it can be just mentioned in the methods, as well as the following paragraph (181-192) describing the Golden Gate approach to prepare the recombinant virus. At least, the paragraph can be shortened.

Response: We have modified accordingly.

Line 377: do not need the reference number of luciferase substrate

Response: We have modified accordingly.

Line 386: for the Golden Gate system, I would appreciate if the authors would precise which region of the region is included in each segment, to allow reproducibility from other groups.

Response: We have now provided the exact cutsite positions in the Supplementary table1.

Line 449: there is a typo of “capable”

Response: We have corrected the typo.
